# Differences in the Clinical Course of Acute Appendicitis in Geriatric Patient Groups

**DOI:** 10.30476/beat.2020.85729

**Published:** 2020-10

**Authors:** Sadettin ER, Sabri Özden, Umut Fırat Turan, Egemen Özdemir, Barış Saylam, Mesut Tez

**Affiliations:** 1 *Department of General Surgery, Numune Training and Research Hospital, Ankara, Turkey*; 2 *Department of Gastrointestinal Surgery, İnönü University School of Medicine, Malatya, Turkey*

**Keywords:** Appendicitis, Mortality, Geriatrics

## Abstract

**Objective:**

To compare the differences in the clinical course of acute appendicitis between early elderly (60-79 years) and late elderly patients (≥80 years).

**Method:**

The sample consisted of 177 patients aged over 60 that underwent surgery at the emergency service with the diagnosis of acute appendicitis between January 2010 and May 2018. Patients’ data were retrospectively obtained from electronic records. Patients that had undergone appendectomy or negative appendectomy or had an appendix tumor were excluded from the study. The patients were divided into two groups by age; early elderly (60-79 years) and late elderly (≥80 years).

**Results:**

Of the 177 patients included in the study, 162(91%) were 60-79 years old and 15 (9%) were over 80. A statistically significant difference was found between the early and late elderly groups in terms of perforation, requirement for intensive care (p =0.001), red cell distribution width (p =0.025), the Clavien-Dindo classification (p =0.020), and the Charlson comorbidity index (p =0.005). The mean hospital stay was four and 11 days for early and late elderly groups, respectively. Multivariate analysis revealed that age alone was an independent factor with a statistically significant effect on mortality (OR=Odds Ratio: 53, 95% CI: 16.91-166.08; (p<0.001)).

**Conclusion:**

In particular, in the elderly patients over 80 years old, the clinical manifestation of acute appendicitis at hospital admission is in the form of perforation. Therefore, in this age group, a careful, precise and prompt diagnosis is crucial.

## Introduction

Acute appendicitis (AA) is one of the most common surgical pathologies in the emergency service [1]. It occurs in around 7% of the general population throughout their life. Approximately 90% of the cases are seen in young people, and only 10% in elderly people aged over 60 years [2]; however, at advanced ages, AA is associated with a very high complication rate and mortality due to delayed diagnosis and treatment in the emergency service or surgical department. Atypical symptoms, delayed diagnosis, perforation, and septic complications are common in geriatric patients at the initial clinical presentation [3, 4]. In the general population, perforation rates range from 20% to 30%, but in the elderly, this rate is considerable increased, varying between 50% and 70% [5, 6]. Studies conducted with elderly AA patients reported high rates of perforation (70%) and morbidity (48%) [5, 7]. In uncomplicated cases, the risk of mortality is <1%, but in the elderly, this rate is within the range of 4% to 8% [4, 8]. Delayed diagnosis is associated with the increase in perforation rate, particularly in this patient group.

In geriatric patients, the most common specific perioperative complications are general complications, such as postoperative wound infection, pneumonia, and cardiovascular problems [9]. The differences in elderly patients compared to other age groups include atypical clinical presentation, acute abdominal causes, such as malignancy other than AA, and secondary peritonitis caused by perforation. Additionally, the operation may be difficult due to atypical presentation and late diagnosis of AA in elderly patients [10]. Especially these patients should have priorities at emergency services due to high perforation risks and mortality, morbidity rates and clinicians should be more precise and quicker in diagnosis. Delay in diagnosis may cause morbidity during the postoperative period and prolong the hospital stay. This study aimed to compare preoperative and postoperative differences in the clinical course of AA between early elderly (60-79 years) and late elderly (≥80 years) patients.

## Material and Methods

*Patients*

This study was planned in Ankara Numune Training and Research Hospital General Surgery clinic. A total of 93 males (52.5%) and 84 females (47.5%) patients over 60 years of age that underwent surgery at the emergency surgery service with the diagnosis of AA between January 2010 and May 2018 were included in the study. Local ethics committee approval has obtained for the study (Approval no: E-18-2118). The patients with AA were retrospectively identified based on the electronic records of the patients. The diagnoses of AA have confirmed by final pathology reports for all 177 patients. Inclusion criteria were, all patients; over 60 years of age, underwent appendectomy between mentioned dates, have confirmation for the diagnosis, and have adequate medical records. Exclusion criteria were having undergone appendectomy and negative appendectomy as part of another surgical procedure or having an appendix tumor. The patients who were confirmed to have undergone appendectomy only due to AA were included in the study. The patients were divided into two groups according to age; early elderly (60-79 years) and late elderly (≥80 years).

*Data*

After obtaining the approval of the ethics committee for the study, the data of 177 patients over 60 years of age obtained from the records were evaluated. The electronic records were screened for details of treatment, surgical notes, and pathology reports. The data obtained from medical records consisted of age, sex, symptoms, physical examination, laboratory findings, imaging, appendicitis type (perforated or non-perforated), postoperative complications, length of hospital stay, and in-hospital mortality. The comorbidities of the patients were examined according to the Charlson comorbidity index (CCI), and their clinical, perioperative and pathologic findings were graded according to the American Association for the Surgery of Trauma (AAST) classification. Postoperative morbidity was defined as presence of wound infections, ileus, intraabdominal abscess or pneumonia complication, and requirement of intensive care. The treatment applied to the complications was classified according to the Clavien-Dindo classification (CDC).

*Statistical analysis*

All statistical analyses were undertaken using SPSS Windows version 12.0 (SPSS Inc., Chicago, Illinois, USA).  The numerical data were given as median (interquartile range; IQR) and compared using the Mann-Whitney U test when the normality assumption was violated. The categorical variables were expressed as percentage (%) and compared with the chi-square or Fisher’s exact tests. Statistically significant variables were assessed by multivariate logistic regression. A *p* value of

## Results

Of the 177 patients included in the study, 162 (91%) were 60-79 years old (early elderly) and 15 (9%) were over 80 years of age (late elderly). There were 87 men (53%) and 75 women (46%) in the former group and six men (40%) and nine women (60%) in the latter group, with the median ages of 66 years (min 60-max: 79) and 85 years (min: 82; max: 93), respectively. A statistically significant difference was found between the early and late elderly groups in terms of perforation (*p*=0.016), intensive care requirement (*p*=0.001), mortality (*p*=0.002), red cell distribution width (RDW) (*p*=0.025), CDC (*p* =0.020), and CCI (p = 0.005). However, there was no statistically significant difference between the two groups in terms of sex, complication (surgical site infection, intra-abdominal abscess, pneumonitis, etc.), white blood cell (WBC) count (10^3^ μl), neutrophil count (10^3^ μl) and AAST grading. The mean hospital stay was four (IQR=2-5) and 11 (IQR=2-6) days in the early and late elderly groups, respectively (*p*=0.154) (Table1). As summarized in Table 1, the multivariate analysis revealed as an independent variable, had a statistically significant effect on mortality (OR: 53, 95% CI: 16.91-166.08; *p*<0.001).

**Table 1:** Distribution of demographic, clinical and laboratory findings in AA patients by age groups and the results of multivariate logistic regression. (n=177).

**Variable**	**Early elderly** **(60-79 years old)** **n=162**	**Late elderly** **(≥80 years old) n=15**	**p value** ^1^	**p value** ^2^
Median age (IQR)	66 (60-79)	85 (82-93)		**<0.001** (OR: 53, 95% CI: 16.91-166.08)
Sex M/F	87/75(53.7/46.3)	6/9(40/60)	0.309	
Perforation	4(2)	10(66)	0.016^a^	0.166
Intensive care requirement	16(9)	6(40)	0.001^ a^	0.890
Mortality	3(2)	3(20)	0.002^ a^	0.527
WBC^f^(10^3 ^/ µl), median (IQR^b^)	13.2(10.5-16.7)	12.2(7.1-15.9)	0.348	
Neutrophil (10^3 ^/ µl), median (IQR^b^)	10.2(7.3-13.9)	8.8(6.3-13.9)	0.490	
RDW^g^(%),median (IQR)	13.6(12.9-14.6)	14.9(13.5-17.3)	0.025^ a^	0.054
CDC^c^ median (IQR)	2(2-2)	5(5-5)	0.020^ a^	0.305
CCI^d^ median (IQR)	4(3-5)	5(4-6)	0.005^ a^	0.504
AAST^e^ median (IQR)	3(1-4)	3(3-4)	0.408	
Hospital stay (mean / days)	4 (IQR: 2-5)	11 (IQR: 2-6)	0.154	
Total	162 (91%)	15 (9%)		

^a^: Statistically significant p <0.005; ^b^IQR: Interquartile range; ^c^ CDC: Clavien-Dindo classification; ^d^ CCI: Charlson comorbidity index; ^e^ AAST: American Association for the Surgery of Trauma; ^f^WBC: White blood cell; ^g^ RDW: Red cell distribution width; p value a : Univariate analysis, p value b : Multivariate analysis; Mann-Whitney U test: Median (interquartile range; IQR) data were evaluated. ; Fisher’s exact test: Percentage expressions were evaluated

## Discussion

AA is one of the most prevalent surgical phenomena in all age groups and is increasingly seen in the elderly population [11]. Geriatric patients tend to have a complex medical history [12]. Clinical presentation of patients in this age group differs on first visit to the hospital. Today, with the increase in life expectancy, AA prevalence in the elderly is growing. Therefore, it is necessary to have a good understanding of the clinical characteristics of this age group and consider AA in the diagnosis of suspected cases. The classical findings of AA, including the triad of fever, leukocytosis and right lower quadrant pain are present in only 10-26% of patients over 60 years of age [13]. Similarly, right lower quadrant pain is not seen in 25% of patients in the advanced age group [14]. Studies have shown that 84% of the elderly consistently use medication, and 46% of these drugs have non-steroidal anti-inflammatory properties, which, when used frequently, suppress pain caused by AA. This results in delayed hospital referral and diagnosis, leading to a higher perforation rate, which is 72% in geriatric patients, as opposed to 20% to 30% in the younger population [15].

In this study, the perforation rate was found to be 2% and 66% in the early and late elderly groups, respectively. This shows that perforation was statistically significantly increased in the patient group aged 80 years or older compared to the patients that were 60 to 79 years old. This higher rate among the late elderly patients can be attributed to their low pain threshold, higher frequency of living alone, presence of multiple co-morbidities, and AA not being considered in initial diagnosis compared to the early elderly patients. Furthermore, the older patients generally have fewer physiological reserves, a higher rate of comorbidities, and a greater possibility of having had previous abdominal surgery, which all increase their surgical risks compared to the younger patient groups.

Tehrani et al. showed that patients who had undergone appendectomy for perforated appendicitis had higher complication and mortality rates than those having had appendectomy without peritoneal closure [16]. Another study reported a mortality rate of 2.3% to 10%, and associated this higher rate particularly with comorbidity, as well as with perforation and septic complications in the elderly [15]. A similar strong relationship was found between CCI and mortality [17]. In the current study, there was a statistically significant difference between the two geriatric groups in terms of CCI (*p* = 0.005). Mortality was also higher in patients aged 80 years and over. However, the 2% mortality rate in the early elderly group underlines the importance of considering AA for early diagnosis and immediate surgical treatment in the over-80 patient group. In this study, the need for postoperative intensive care in the late elderly group was found to be statistically significant. This may be associated with comorbidities or a result of late diagnosis and perforation.

It has been reported that some surgeons hesitate to perform surgery on octogenarians and choose to monitor them until their symptoms become more pronounced, but this delays diagnosis and increases the possibility of perforation [18]. Other reasons for delayed treatment of AA in elderly patients have been suggested as abdominal muscle atrophy, loss in the nervous system functions related to sensing, conduction and assessment of pain, comorbid conditions (e.g., hearing loss, dementia, cerebrovascular disease, Alzheimer’s disease, and psychiatric problems), and regular and simultaneous use of several drugs [6]. Another challenge is the communication difficulties experienced by some octogenarian patients [19] that result in these patients misremembering or not recalling the onset of their symptoms. All these factors increase the possibility of delayed diagnosis, morbidity, and mortality.

In the literature, age was shown to have an independent effect on mortality and postoperative morbidity [20]. Blomqvist et al., [2] also reported that the mortality rate was strongly related to age. There are also studies suggesting that advanced age increases [21] or does not affect [22] mortality in comparisons between geriatric groups. In another study, age was found as an independent indicator of mortality, especially among the octogenarians [23]. Similarly, we determined that age alone was an independent factor affecting mortality (OR: 53%, CI: 16.91-166.08; *p* = 0.000).

Current studies indicate that elderly patients have a longer hospital stay than younger patients following appendectomy, and the average of this duration is longer than one week [24]. In this study, the length of hospital stay was four and 11 days in the early elderly and late elderly patients, respectively; however, there was no statistically significant difference between the two groups.

Geriatric patient groups are usually difficult to manage and require a multidisciplinary approach in terms of diagnosis and treatment. A highly sensitive and effective diagnostic test for AA is very desirable in elderly patients in order to avoid high risks associated with surgery and possible complications due to delayed diagnosis. In the literature, RDW has been shown as a good indicator of systemic inflammation and mortality in elderly patients [25]. The RDW rate in the current study was statistically significantly higher in patients aged 80 and over than those aged 60 to 79 years.

Huive *et al*., [26] detected abscess formation in 38% of the elderly AA patients they evaluated. In the group over 80 years of age in the current study, the median CCI and CDC were both 5 (4-6 and 5-5, respectively). In the majority of these patients, mortality developed before postoperative complications due to advanced age and increased CCI. In addition, many patients in this group required intensive care.

The limitation of this study includes the retrospective design, and consequently the data being based on existing electronic records of the patients.

In conclusion, the anamnesis of elderly patients is often incomplete, complex, and atypical or has different clinical features and multiple comorbidities. Especially in the octogenarian and nonagenarian patient groups, the presentation of AA in clinical practice is in the form of perforation. Therefore, these patients’ groups require a more careful, meticulous and rapid diagnosis.
